# A Phase I Study of Nimotuzumab With Nivolumab in Advanced Non-small Cell Lung Cancer and Head and Neck Squamous Cell Cancer

**DOI:** 10.7759/cureus.98843

**Published:** 2025-12-09

**Authors:** Syed Ather Hussain, Mary E Reid, Rachel Frascati, Adrienne Groman, Amparo Macias, Patricia L Luaces, Orestes Santos-Morales, Kalet Leon, Tania Crombet, Grace K Dy

**Affiliations:** 1 Department of Thoracic Medicine, Roswell Park Comprehensive Cancer Center, Buffalo, USA; 2 Department of Medicine, Roswell Park Comprehensive Cancer Center, Buffalo, USA; 3 Clinical Trials Office, Roswell Park Comprehensive Cancer Center, Buffalo, USA; 4 Department of Biostatistics, Roswell Park Comprehensive Cancer Center, Buffalo, USA; 5 Department of Therapeutics, Centro de Inmunologia Molecular, Havana, CUB

**Keywords:** anti-epidermal growth factor receptor, anti-programmed cell death protein 1, head and neck squamous cell cancer, monoclonal antibody, non-small cell lung cancer

## Abstract

Introduction: Nimotuzumab is a monoclonal antibody (mAb) that targets the epidermal growth factor receptor. Nivolumab is an anti-programmed death 1 (anti-PD1) mAb that overcomes immune checkpoint inhibition. This trial aimed to evaluate the safety, tolerability, and efficacy of combination treatment as second-line therapy in non-small cell lung cancer (NSCLC) and head and neck squamous cell cancer (HNSCC).

Methods: This was a single-center, single-arm, non-randomized study utilizing a three plus three dose escalation design. Nimotuzumab was given intravenously (IV) at escalating doses of 200 mg, 300 mg, and 400 mg after nivolumab infusion at 240 mg IV every two weeks in a 28-day cycle. Recommended phase 2 dose (RP2D) and dose-limiting toxicities (DLT) were determined. Treatment was continued until disease progression, unacceptable toxicity, or withdrawal from the trial. Serial EKG and troponin checks were performed.

Results: Seven patients were enrolled across the 200 mg and 300 mg nimotuzumab dose levels. The median age was 64.1 years. Four patients (57.1%) were males. Five (71.4%) had NSCLC, and the predominant histology was pure adenocarcinoma (n = 4, 57.1%). Four patients (57.1%) had Kirsten rat sarcoma (KRAS) mutation. All patients had disease progression after platinum-based and anti-PD1 therapy prior to enrollment, except for two NSCLC patients who were enrolled to receive this treatment after initial platinum-based chemotherapy only. Four patients (two from each dose level) had troponin elevation (median = 0.1 ng/ml, range = 0.07 to 0.3 ng/ml) lasting at least 24 hours, which normalized next week without any associated chest pain, except for one patient (HNSCC) with potential treatment-related DLT. Disease control rate was 42.9% (stable disease as best response, n = 3).

Conclusions: Asymptomatic troponin elevation was seen with this combination treatment. While there were no clinically significant adverse cardiac outcomes, further development was terminated due to the competitive landscape.

## Introduction

Lung cancer is the leading cause of cancer-related mortality worldwide [1]. Of all cases of lung cancer, 85-90% are categorized as non-small cell lung cancer (NSCLC), and 70% are either locally advanced or metastatic at the time of disease presentation [2]. Despite great strides in recent years, lung cancer prognosis remains dismal, with five-year overall survival (OS) rates of only 17.4% and less than 5% in the metastatic setting [3].

Historically, chemotherapy with a platinum-doublet has resulted in a median OS of about 12 months for patients with advanced NSCLC [4]. The advent of targeted therapy has led to significant OS improvements, but only in selected patient populations harboring the corresponding specific genetic alterations. Additionally, immunotherapy has become the first-line treatment option for advanced NSCLC, either as monotherapy in case of high programmed death-ligand 1 (PD-L1)-expressing tumors (>50%) or in conjunction with platinum-based chemotherapy regardless of the PD-L1 status [5]. Despite all these notable advancements, lung cancer prognosis remains poor, warranting the need to develop innovative therapeutic options and combination treatments.

Epidermal growth factor receptor (EGFR) is overexpressed in several different human tumors, including NSCLC and head and neck squamous cell cancers (HNSCC) [2]. Nimotuzumab is a humanized IgG1 monoclonal antibody (mAb) used in Asia for the treatment of HNSCC, which targets the extracellular domain III of EGFR, hence impeding epidermal growth factor (EGF) binding and sterically preventing receptor dimerization, resulting in decreased cellular proliferation, angiogenesis, pro-apoptotic signals, and antibody-dependent cellular cytotoxicity (ADCC) [2]. Nimotuzumab binding is heavily dependent on cell receptor density, which means the mAb binds high EGFR-expressing tumor cells with increased avidity, whilst at the same time sparing low EGFR-expressing normal cells, hence avoiding unnecessary adverse effects, such as skin toxicity, which is frequently seen with other EGFR mAbs, all without compromising on efficacy [6].

Nivolumab is an anti-programmed cell death protein 1 (PD-1) mAb, which overcomes immune checkpoint inhibition and is approved for advanced NSCLC and HNSCC. Nivolumab not only enhances T-cell effector function, including cytolytic activity against tumor cells, but also promotes infiltration of tumor-reactive CD8+ T cells into the tumor microenvironment [7].

Preclinical studies have shown that activation of the EGFR pathway leads to the upregulation of immune checkpoint molecules such as PD-L1 on tumor cells, which consequently allows them to evade immune-mediated cytotoxicity [8,9]. We thus hypothesized that signaling through EGFR may potentially mediate resistance to immunotherapy. Given their complementary mechanism of action, the nimotuzumab/nivolumab combination treatment was speculated to provide synergistic anti-tumor response, requiring further investigation. This trial aimed to evaluate the safety, tolerability, and efficacy of nimotuzumab/nivolumab combination treatment for advanced NSCLC and HNSCC (NCT02947386).

This article was previously presented as a meeting abstract at the 2024 World Conference on Lung Cancer on September 7, 2024 [10].

## Materials and methods

Patient eligibility

Patients with pathologically confirmed unresectable NSCLC or HNSCC who received prior treatment with platinum-based doublet chemotherapy with or without immunotherapy were eligible for the study. Other inclusion criteria included age ≥18 years, an Eastern Cooperative Oncology Group (ECOG) performance score of at most 2, candidacy for immunotherapy, at least six months of life expectancy, adequate bone marrow reserve and organ function, and presence of evaluable disease for the phase 1 portion of the study. The planned phase 2 component of the original study did not accrue any patients, as the study was terminated early due to poor accrual related to rapid evolution in the standard of care.

Patients with autoimmune disorders, a history of invasive cancer within the last two years, active clinically serious infection requiring treatment, radiotherapy within two weeks prior to starting study treatment, active/untreated brain metastasis, leptomeningeal involvement, major surgery within 14 days prior to starting study drug, clinically significant interstitial lung disease, or immunodeficiency were excluded from the study. Patients who had received chemotherapy, immunotherapy, or anti-EGFR antibody within four weeks before the first administration of the study drug or those who had known hypersensitivity to any of the components of the study drugs or their analogues were also not eligible for the study.

Written informed consent was obtained from all patients before study registration. The study was approved by the Institutional Review Board of Roswell Park Comprehensive Cancer Center and was conducted according to the Declaration of Helsinki and the Good Clinical Practice guidelines. This clinical trial has been registered at ClinicalTrials.gov. The trial registration number is NCT02947386. The date of registration is 10/26/2016.

Trial design

This was a single-center, single-arm, non-randomized study utilizing a conventional three plus three dose escalation design. Nimotuzumab was given intravenously (IV) at planned escalating doses of 200 mg, 300 mg, and 400 mg every two weeks in sequence to nivolumab infusion at 240 mg IV every two weeks in a 28-day cycle.

The phase 1 portion of the trial aimed to examine dose-limiting toxicities (DLTs) and to estimate the maximum tolerated dose (MTD) of nimotuzumab and nivolumab combination to use as the recommended phase 2 dose (RP2D) in the treatment of advanced NSCLC or HNSCC.

A DLT was defined as the occurrence of any of the following events within the first treatment cycle, which was considered by the investigator to be possibly related to either of the study drugs: grade 4 neutropenia, grade 3 or above febrile neutropenia, grade 4 thrombocytopenia, grade 3 thrombocytopenia with bleeding, grade 4 treatment-related rash or any other grade 3 or above non-hematologic toxicity. The DLT period was defined as the first cycle of combination therapy (i.e., completion of two doses of nivolumab and nimotuzumab). DLT-evaluable patients were those who had a DLT or who completed the DLT period without a DLT.

The MTD was defined to be the highest dose level at which at least one out of six patients had DLTs. Six DLT-evaluable patients had to be treated at a dose level for it to be declared the MTD. Hence, no more than one DLT should occur in a minimum of six patients enrolled at a dose level to adequately assess safety for the determination of RP2D. Toxicities were assessed by the investigator using the United States (US) National Cancer Institute (NCI) Common Terminology Criteria for Adverse Events (CTCAE), version 4.0.

Assessment plan

At baseline, all patients had a complete history and physical exam done, including assessment of ECOG performance status, along with complete blood count (CBC) with differential, complete metabolic panel (CMP), thyroid function tests, cardiac labs (troponin, beta natriuretic peptide, creatine kinase total, and creatine kinase-MB), coagulation parameters, urinalysis, 12-lead electrocardiogram (EKG), echocardiogram, computed tomography (CT) scan of chest, abdomen, and pelvis, and magnetic resonance imaging (MRI) of the brain with contrast.

Tumor was restaged via CT scan on cycle three, day one (+/- a week) and on day one (+/- a week) of every two cycles thereafter. MRI of the brain was repeated only as clinically indicated. A CT scan of the brain with contrast was an acceptable alternative if there was a contraindication or if the patient was unable to undergo a brain MRI. Cardiac labs and EKG were performed weekly for the first three cycles, then on cycle six, day one, and then as clinically indicated.

Tumor response was assessed according to RECIST 1.1 (Response Evaluation Criteria in Solid Tumors) as mentioned previously. The frequency of adverse events (AEs) was tabulated by grade across all dose levels and cycles. All patients who received any study treatment were considered evaluable for toxicity.

Statistical analysis

Baseline characteristics, toxicity, and efficacy were analyzed with descriptive statistics. Microsoft Excel spreadsheet version 16.101.3 (Microsoft Corporation, Redmond, WA) was used for analysis.

## Results

Patient characteristics

Seven patients with metastatic NSCLC (n = 5) and HNSCC (n = 2) were recruited in this study from April 2018 to March 2021. The baseline characteristics of all patients are summarized in Table 1. The median age was 64.1 years (range = 50.1-79.4 years). Four patients (57.1%) were males, and most patients were of White ethnicity (n = 6, 85.7%). The majority of patients with NSCLC had adenocarcinoma (n = 4) and Kirsten rat sarcoma (KRAS) gene mutation (n = 4). All patients had disease progression after platinum-based and anti-programmed death 1 (anti-PD1) therapy prior to enrollment, except for two NSCLC patients who were enrolled to receive this treatment after initial platinum-based chemotherapy only.

**Table 1:** Demographics. *HNSCC patient: no mutation profiling results available. HNSCC: head and neck squamous cell cancer.

	Nimotuzumab 200 mg	Nimotuzumab 300 mg	Overall
Patients enrolled (n, %)	3 (42.9%)	4 (57.1%)	7 (100%)
Age (Median, range)	64.1 (57.1-74.2)	61.4 (50.1-79.4)	64.1 (50.1-79.4)
Male	2 (66.7%)	2 (50.0%)	4 (57.1%)
Race			
White	3 (100.0%)	3 (75.0%)	6 (85.7%)
Unknown/Others	0 (0%)	1 (25.0%)	1 (14.3%)
Histology			
Adenocarcinoma	3 (100.0%)	2 (50%)	5 (71.4%)
HNSCC, p16 positive	0 (0%)	2 (50.0%)	2 (28.6%)
KRAS mutation status (n, %)			
Wildtype	1 (33.3%)	1 (25%)	2 (28.6%)
KRAS G12C	1 (33.3%)	0 (1%)	1 (14.3%)
Other KRAS variants	1 (33.3%)	2 (50%)	3 (42.8%)
Unknown*	0 (0%)	1 (25%)	1 (14.3%)
Performance status (n, %)			
0	1 (33.3%)	1 (25%)	2 (28.6%)
1	2 (66.7%)	3 (75%)	5 (71.4%)

Toxicity and safety assessment

Three patients received the 200 mg nimotuzumab dose without DLTs, and four patients received the 300 mg dose in combination with nivolumab, one of whom had a potential treatment-related DLT. However, since the accrual stopped early, the safety of the 400 mg nimotuzumab dose in combination with nivolumab remains undetermined.

All seven patients were evaluable for toxicities. Common treatment-emergent toxicities are summarized in Table 2. There were no treatment-related grade 4 or 5 toxicities. The most common AE was grade 1 and 2 nausea, experienced by six patients (85.7%), followed by grade 1 fatigue, which was experienced by five patients (71.4%). Four patients (57.1%), two from each dose level, had troponin elevation (median = 0.1 ng/ml, range = 0.07-0.3 ng/ml). Troponin elevation was documented in more than one measurement for three of the four patients, lasting at least 24 hours, which normalized the following week without any associated chest pain or other cardiovascular event to resume dosing, except for one patient (HNSCC) at the nimotuzumab 300 mg dose level. This patient was considered to have encountered potential treatment-related DLT, given grade 3 troponin elevation (0.14 ng/ml) documented on day eight assessment, when the patient also reported new-onset anginal-type pain lasting about two days, starting within 24 hours after completing cycle one, day one treatment dose. No EKG or echocardiogram changes were noted during the cycle one, day eight evaluation. Repeat troponin on cycle one, day eight, showed persistent elevation above normal (0.09 ng/ml). This patient underwent cardiac catheterization for further evaluation and demonstrated mild non-obstructive atherosclerotic disease. Other common low-grade treatment-emergent AEs included chills (n = 4, 57.1%), weight loss (n = 4, 57.1%), low appetite (n = 3, 42.9%), constipation (n = 3, 42.9%), arthralgias (n = 3, 42.9%), asthenia (n = 3, 42.9%), and dizziness (n = 3, 42.9%).

**Table 2:** Common treatment-emergent adverse events.

System organ class	Preferred term	Total (N = 7)				
		Grade, N (%)				
		Grade	Grade	Grade	Grade	Total
		1	2	3	5-Apr	
	Abdominal pain	1 (14.3)	1 (14.3)	0 (0.0)	0 (0.0)	2 (28.6)
	Constipation	3 (42.9)	0 (0.0)	0 (0.0)	0 (0.0)	3 (42.9)
	Diarrhea	2 (28.6)	0 (0.0)	0 (0.0)	0 (0.0)	2 (28.6)
	Nausea	5 (71.4)	1 (14.3)	0 (0.0)	0 (0.0)	6 (85.7)
General disorders and administration site conditions	Asthenia	3 (42.9)	0 (0.0)	0 (0.0)	0 (0.0)	3 (42.9)
	Chills	3 (42.9)	1 (14.3)	0 (0.0)	0 (0.0)	4 (57.1)
	Early satiety	3 (42.9)	0 (0.0)	0 (0.0)	0 (0.0)	3 (42.9)
	Fatigue	5 (71.4)	0 (0.0)	0 (0.0)	0 (0.0)	5 (71.4)
	Edema peripheral	2 (28.6)	0 (0.0)	0 (0.0)	0 (0.0)	2 (28.6)
Investigations	Troponin increased	1 (14.3)	2 (28.6)	1 (14.3)	0 (0.0)	4 (57.2)
	Weight decreased	3 (42.9)	1 (14.3)	0 (0.0)	0 (0.0)	4 (57.1)
	Decreased appetite	2 (28.6)	1 (14.3)	0 (0.0)	0 (0.0)	3 (42.9)
Metabolism and nutrition disorders	Arthralgia	3 (42.9)	0 (0.0)	0 (0.0)	0 (0.0)	3 (42.9)
Musculoskeletal and connective tissue disorders	Muscle spasms	2 (28.6)	0 (0.0)	0 (0.0)	0 (0.0)	2 (28.6)
	Musculoskeletal pain	1 (14.3)	1 (14.3)	0 (0.0)	0 (0.0)	2 (28.6)
	Dizziness	3 (42.9)	0 (0.0)	0 (0.0)	0 (0.0)	3 (42.9)
	Anxiety	0 (0.0)	1 (14.3)	0 (0.0)	0 (0.0)	1 (14.3)
Psychiatric disorders	Cough	2 (28.6)	0 (0.0)	0 (0.0)	0 (0.0)	2 (28.6)
Respiratory, thoracic, and mediastinal disorders	Dyspnea	1 (14.3)	1 (14.3)	0 (0.0)	0 (0.0)	2 (28.6)
	Pleural effusion	0 (0.0)	1 (14.3)	1 (14.3)	0 (0.0)	2 (28.6)

Efficacy assessment

Of the seven patients enrolled and who received study treatment, one was not evaluable for response, as this patient was deemed to potentially have DLT due to chest pain and grade 3 troponin elevation after only receiving one dose of study treatment and was taken off study. Objective tumor response in the six response-evaluable patients included three stable disease (SD) and three progressive disease (PD). All three patients with SD had NSCLC, two of whom had low PD-L1 expression (1-49%), STK11 wildtype but KRAS-mutated adenocarcinoma, and were previously treated with an immune checkpoint inhibitor. The third NSCLC patient with SD was immune checkpoint inhibitor-naïve, had PD-L1 negative adenocarcinoma, and no KRAS/STK11/KEAP1 alteration was seen. The other immune checkpoint inhibitor naïve NSCLC patient, who had PD on study treatment, had a KRAS/STK11-mutated PD-L1-negative lung adenocarcinoma. One of the two HNSCC patients enrolled had PD as the best response (the other patient had the DLT and received only one dose). Overall disease control rate (DCR) based on intention-to-treat analysis was 42.8% (three out of seven patients). In summary, among those with SD, two patients were PD-L1 positive, while among those with PD, two patients had a KRAS mutation. The median progression-free survival (PFS) was found to be two months (95% CI: 1.8-3.8), and the median OS was found to be seven months (95% CI: 1.9-15.3).

## Discussion

A French trial from 2020 investigated the efficacy and safety of a different EGFR mAb, necitumumab, in combination with pembrolizumab for stage IV NSCLC in the second-line setting after progression on platinum-based chemotherapy or targeted therapy [11]. This study reported an overall response rate (ORR) of 23.4% (95% CI: 13.8%-35.7%) in the immune checkpoint inhibitor-naïve NSCLC population. Amongst all patients, two (3.1%) had a complete response (CR), 13 (20.3 %) had a partial response (PR), 26 (40.6%) had SD, 17 (26.6%) had PD, and six (9.4%) were not evaluable [11]. Irrespective of the tumor histology or PD-L1 status, this trial reported modest benefits with a median PFS of 4.1 months (95% CI: 2.4-6.9 months) and a six-month OS rate of 74.7% (61.5%-83.9%). DCR was found to be 64.1% (95% CI: 51.5%-75.7%). In the French study, upon subgroup analysis, patients who were PD-L1 positive had a better ORR (40.0% for strong positive status vs. 25.0% for weak positive status), as well as an improved median PFS (7.6 months for strong positive status vs. 5.4 months for weak positive status) in contrast to patients with PD-L1-negative tumor (12.5% and 2.7 months, respectively) [11]. While our study had a lower response rate, median PFS, and DCR, it is to be noted that the majority of our patients had either primary or acquired resistance to immunotherapy, in contrast to the French study, which recruited an immune checkpoint inhibitor-naïve population. Yet, we observed a similar six-month OS rate of 71% (26%-92%).

Our study results contrast with the French study for several reasons. Chiefly, our patient population was more heavily pre-treated, as most patients had previously received platinum-based chemotherapy as well as immunotherapy in earlier lines of treatment, whereas the French study enrolled patients who were immunotherapy-naïve. Secondly, the majority of NSCLC patients enrolled harbored a KRAS mutation. For NSCLC, the role of KRAS mutations as a prognostic or predictive marker remains controversial, likely due to heterogeneous outcomes related to the variable impact of co-mutations. Recent data demonstrate that concurrent pathogenic mutations in STK11 or KEAP1 are associated with diminished treatment responses and worse survival outcomes [12]. In colon cancer, KRAS mutation has been identified as a strong negative predictive biomarker that confers resistance to EGFR inhibitors [13]. It is possible that our study did not show a benefit to combination treatment due to a sizable number of KRAS-mutated tumors.

More recently, an abstract presented at the American Association for Cancer Research (AACR) 2024 meeting reported outcomes for 50 patients who had received pembrolizumab and necitumumab combination treatment in the first-line setting for advanced NSCLC with PD-L1 expression of more than 50% [14]. The primary analysis showed that the ORR was 76.0% (95% CI: 61.9%-86.9%); 2.0% had a CR, 74.0% had a PR, 10.0% had SD, and 8.0% had PD. The median PFS was 15.7 months (95% CI: 10.3 to not reached), and the median OS was not reached [14]. The PFS and relative risk (RR) observed in this cohort of patients from Japan seem promising and, on the surface, better than what has been observed in larger global phase III studies of anti-PD1/PD-L1 agent monotherapy in this population [15]. Further investigation to confirm this is required, given the limitations of cross-trial comparisons and heterogeneity in patients enrolled across trials. Nonetheless, this study highlighted that patients with PD-L1-high tumors, who received combination treatment in an earlier line setting, may potentially have better outcomes compared to their use in a later line setting.

In terms of toxicity profile, three patients (42.9%) had asymptomatic troponin elevation lasting at least 24 hours occurring during the week of their scheduled dose three or four, while one patient (14.3%) had potential treatment-related troponin elevation with associated cardiac symptoms on cycle one, day eight, necessitating treatment cessation and further interventions. In a prospective study characterizing the role of troponin in assessing cardiac toxicity from nivolumab among NSCLC patients, troponin elevation above the normal range was seen in 24% (inclusive of 1.7% with elevated troponin at pre-treatment baseline). None of these patients had a cardiovascular event during the observation period [16]. The observed frequency of troponin elevation in our study is much higher than expected, raising the question of the contribution of nimotuzumab. While cardiac events have not been described for nimotuzumab previously, earlier studies did not mandate routine monitoring of troponin levels, in contrast to our study. Indeed, elevation of troponin has been observed in a prospective study of colorectal cancer patients with normal baseline troponin among 37% patients receiving the EGFR antibodies cetuximab or panitumumab. There were no severe cardiovascular events in this group, similar to what we observed. We thus postulate that the observed asymptomatic troponin elevation is most likely related to the class effect of EGFR antibodies, with a contribution from the underlying disease state of individuals enrolled.

The main limitation of this study was its small sample size, which was not powered to reach definitive conclusions. Another limitation was that both NSCLC as well as HNSCC patients were grouped into a single cohort. Inferential statistics could not be used reliably, given the small sample size, which is a shortcoming of this study. Additionally, the accrual for the study stopped early due to rapidly changing treatment options, and thus, the safety of the 400 mg nimotuzumab dose in combination with nivolumab could not be determined.

## Conclusions

Given the lack of an early efficacy signal in pursuing this combination in the pre-treated NSCLC population, as well as the competitive landscape in drug development, this trial was terminated prematurely, and nimotuzumab/nivolumab combination treatment is no longer being developed for this patient population. However, our study did help to further improve our understanding of EGFR inhibitors and their adverse effect profile. While the findings must be interpreted with some caution due to the small sample size, our study observed asymptomatic troponin elevation as a potential new treatment-related adverse event that can be observed with nimotuzumab, which could be a class effect of EGFR inhibitors.
